# Thiolated Hyaluronic Acid as Versatile Mucoadhesive Polymer: From the Chemistry Behind to Product Developments—What Are the Capabilities?

**DOI:** 10.3390/polym10030243

**Published:** 2018-02-28

**Authors:** Janine Griesser, Gergely Hetényi, Andreas Bernkop-Schnürch

**Affiliations:** 1 Thiomatrix Forschungs-und Beratungs GmbH, Trientlgasse 65, 6020 Innsbruck, Austria; j.griesser@thiomatrix.com (J.G.); hetenyig@gmail.com (G.H.); 2 Center for Chemistry and Biomedicine, Department of Pharmaceutical Technology, Institute of Pharmacy, University of Innsbruck, Innrain 80/82, 6020 Innsbruck, Austria

**Keywords:** thiolated hyaluronic acid, hydrogel, mucoadhesive, biocompatibility, controlled release, drug delivery, wound healing

## Abstract

Within the last decade, intensive research work has been conducted on thiolated hyaluronic acids (HA-SH). By attaching sulfhydryl ligands onto naturally occurring hyaluronic acid various types of HA-SH can be designed. Due the ability of disulfide bond formation within the polymer itself as well as with biological materials, certain properties such as mucoadhesive, gelling, enzyme inhibitory, permeation enhancing and release controlling properties are improved. Besides the application in the field of drug delivery, HA-SH has been investigated as auxiliary material for wound healing. Within this review, the characteristics of novel drug delivery systems based on HA-SH are summarized and the versatility of this polymer for further applications is described by introducing numerous relevant studies in this field.

## 1. Introduction

Hyaluronic acid (HA) is a polysaccharide exhibiting a linear molecular shape. The basic structure consists of two repeating saccharide units, namely d-glucuronic acid and *N*-acetyl glucosamine [1]. Over the years numerous modifications of HA have been introduced. Among them thiolated HA seems to be the most promising one. The attachment of ligands containing free thiol groups onto the polymeric backbone of HA has been the first time described by Bernkop-Schnürch [2] and opens wide possibilities for pharmaceutical applications by tailoring its function. Through thiolation of HA properties such as mucoadhesiveness, swelling capacity, stability and biocompatibility could be improved [3]. The protection of thiol groups via crosslinking or preactivation forming disulfide bonds generates more stable and effective drug delivery systems as the properties of thiomers are further improved [4]. Apart from drug delivery systems, thiolated HA is modified through crosslinkage leading to a scaffold structure and could be consequently applied for wound healing and tissue engineering. Within ocular regenerative medicine, for instance, an improved coronal and ocular wound care is highly on demand and the application of thiolated HA might be a promising approach [5].

It is the aim of this review to provide a comprehensive overview regarding the capabilities of thiolated HA starting with its properties—concretely, mucoadhesive properties, gelation properties, stability, enzyme inhibition properties, biocompatibility, permeation enhancing properties and controlled release. Moreover, further applications, various drug delivery systems (buccal, vaginal, and ocular) and finally product developments are discussed.

## 2. The Chemistry Behind

**Figure 1:**
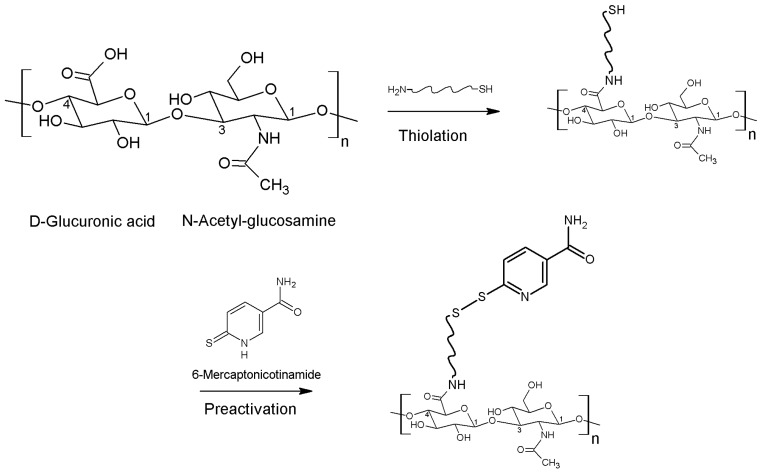
Chemical structure of disaccharide unit within the polysaccharide HA and schematic illustration of thiolation via amidation as well as preactivation with 6-mercaptonicotinamide as an example resulting in a disulfide bond.

The linear polysaccharide HA consists of disaccharide units namely d-glucuronic acid and *N*-acetyl glucosamine linked via β(1,4) and β(1,3) glucosidic bonds, as illustrated in Figure 1. Due to the hydrophilicity of HA, it forms hydrogen bonds between water molecules and carboxyl- as well as acetyl-groups. As HA exhibits a high molecular weight and strong interactions with water, HA is highly viscous in aqueous solution [6,7]. Furthermore, HA naturally occurs in human body from the extracellular matrix of connective tissue, across the dermis of the skin and up to the vitreous body of the eye. Therefore, the major role of HA within the body is giving structure to tissues as well as hydration [8,9]. With a biological half-life of maximal 24 h, HA is continuously degraded within the organism by hyaluronidase enzymes as well as via HA cell internalization by CD44 cell surface receptors [10,11]. Apart from enzymatic degradation, HA could be cleaved through many ways such as via acidic or alkaline hydrolysis, thermal degradation and degradation by oxidants [12]. Due to high water solubility, the development of polymers used for tissue engineering represented a problem [13]. Hence, chemical modification and crosslinking is mandatory in order to expand the degradation time as well as to improve the mechanical stability in vivo [14]. Two chemical groups, namely carboxylic and hydroxyl groups, can be modified via different chemical reactions resulting in thiolated HA, as listed in Table 1. Moreover, Figure 1 illustrates the thiolation of carboxylic groups in the first step and subsequently the protection of this thiol group via preactivation utilizing 6-mercaptonicotinamide to provide just one example. Via this preactivation with 6-mercaptonicotinamide the free thiol groups are oxidized to disulfide bonds. As free thiol groups from thiolated HA are sensitive towards oxidation and rapidly react with the cysteine-rich domains in the mucus, S-protection (=preactivation) plays a major role in the improvement of mucoadhesiveness, cohesiveness as well as stability against degradation [4].

## 3. Properties of Thiolated Hyaluronic Acid

### 3.1. Mucoadhesive Properties

**Figure 2:**
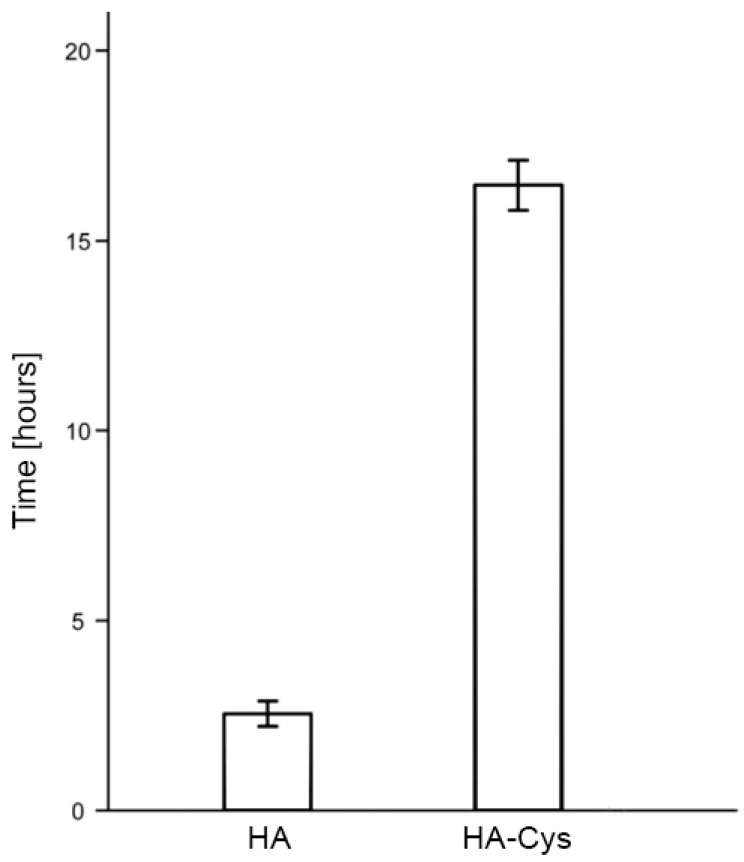
Adhesion time in hours of unmodified HA and HA-Cys on the rotating cylinder in 0.1 M phosphate buffer pH 6.8 with 1% NaCl at 37 °C. Indicated values are means of at least three experiments (±SD). Adapted from Kafedjiiski et al. [14].

The improved mucoadhesive properties of thiolated hyaluronic acid (HA-SH) are primary based on the disulfide bonds between the sulfhydryl moieties of the polymer backbone and the cysteine-rich residues of the mucus. On the contrary, unmodified mucoadhesive polymers are only capable of forming ionic interactions, hydrogen bonds and electrostatic bonds with the mucosal surface [63]. In order to compare and evaluate in vitro mucoadhesiveness of unmodified and modified polymers, the rotating cylinder method represents one of the most studied and feasible methods [64]. For instance, Kafedjiiski et al. showed a 6.5-fold prolonged adhesion time comparing HA-cysteine ethyl ester (HA-Cys) with HA applying the rotating cylinder method (Figure 2) [14]. Recently, Laffleur et al. [23] demonstrated that thiolation of HA leads to an even 12-fold augmentation in mucoadhesion on buccal mucosa. Thiolated HA preactivated with 6-mercaptonicotinamide demonstrated a 4-fold improved adhesion time compared to thiolated HA [19]. These findings are based on the formation of covalent bonds between thiol groups of thiolated HA and cysteine-rich domains within the mucin glycoproteins [65,66]. Besides, Ding et al. [27] developed a delivery system for insulin, namely, multilayered mucoadhesive hydrogel films with thiolated HA and polyvinyl alcohol leading to unidirectional controlled insulin release.

### 3.2. Cohesive Properties

**Figure 3:**
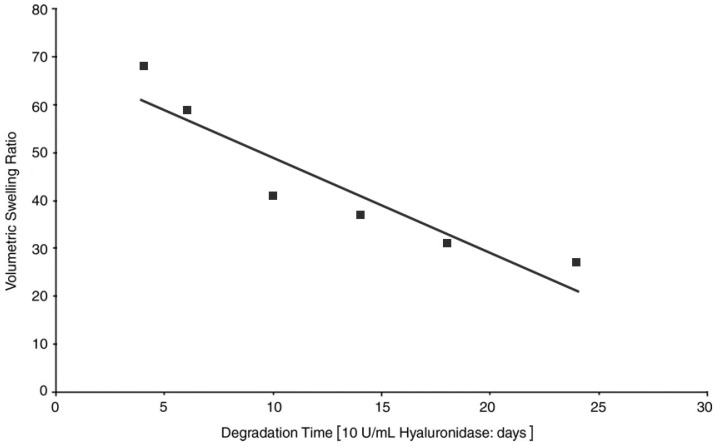
Comparison of swelling ratio and degradation time for six selected chemically modified thiolated HA variants. Increased swelling correlated with rapid degradation. Adapted from Orlandi et al. [46].

Swelling capacity has a major impact on adhesive and cohesive properties of thiolated HA. Generally, swelling capacity is determined by measuring the water uptake in function of time. Numerous research groups confirmed that HA-SH possesses superior properties over HA in terms of water uptake. For example, HA-cysteine ethyl ester exhibited 3.5-fold improved swelling capacity compared to the unmodified polymer [23]. Laffleur et al. [19] mentioned recently that it is not mandatory to reach the maximum swelling capacity in order to take the advantage of thiolated polymers. On the contrary, to overcome the worrying bottleneck of biodegradability, a balance between swelling and stability should be guaranteed. As through the rapid swelling the surface of the polymer increases and as a result, the degradable area for hyaluronidases grows. For instance, Figure 3 illustrates the close relation between swelling ratio and degradation rate. In brief, the more swelling of the polymer could be observed, the faster the hydrogel was degraded, as the hydrogel surface was increased through the swelling process. Thus, enzymes such as hyaluronidase could cleave HA into its fragments of d-glucuronic acid and *N*-acetyl-d-glucosamine in a faster manner [46].

As the gel stiffness also has an impact onto the cohesive properties, Vanderhooft et al. [47] performed rheological measurements of crosslinked thiolated HA gelatin hydrogels showing that the composition as well as the crosslinking rate plays a major role in the gel stiffness properties. This strategy might be useful to tailor polymer properties in consideration of application sites. Moreover, disulfide-crosslinked hyaluronan-gelatin sponges showed growth of fibrous tissue in mice representing high stiffness and a promising approach for tissue augmentation [40]. As gel stiffness represents an important parameter for tissue engineering in order to choose the appropriate composition considering the right tissue, Horkay et al. [51] investigated the structural, mechanical and osmotic properties of injectable hydrogels with respect of the polymer concentration and composition. The concentration of carboxymethylated thiolated HA showed a higher impact onto elasticity compared to the crosslinking density of the polymer backbone.

Overall, it could be discovered that the thermodynamic properties are mainly determined by the polymer concentration and other parameters such as interactions between the two polymeric compounds merely contribute to the thermodynamic behavior of the gels. Bian et al. [26] could verify that gelation time, swelling properties and controlled degradation behavior show correlation on the composition of HA-SH polymers.

### 3.3. Stability against Degradation

**Figure 4:**
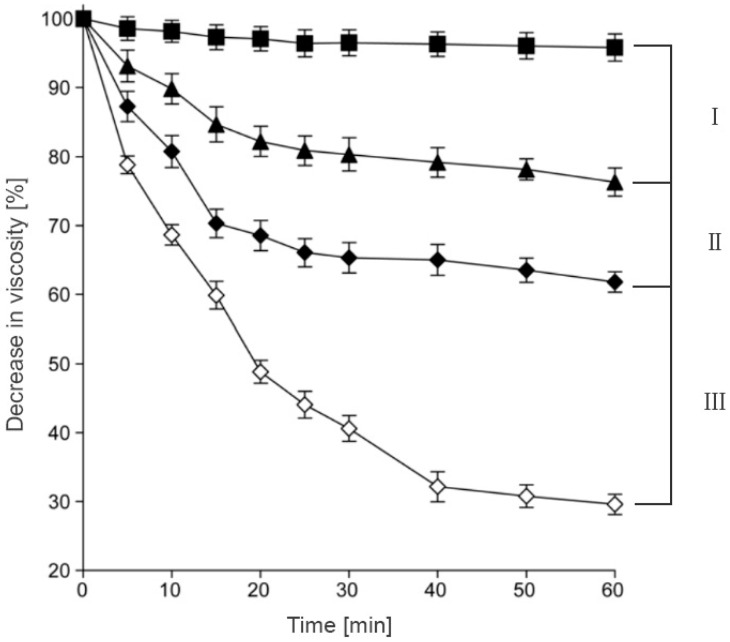
Decrease in viscosity of HA and HA-Cys by hyaluronidase (0.5 mg/mL) at pH 6.0; HA without hyaluronidase (■), HA-Cys (crosslinked) (▲), HA-Cys (♦) and HA (◊). Indicated values are means of at least three experiments (±SD); I and II differ from III, *p* < 0.00001; I, differs from II, *p* < 0.0006. Adapted from Kafedjiiski [14].

As mentioned previously, swelling capacity is important for the mucoadhesiveness of thiolated HA. Nevertheless, its stability is negatively influenced by pronounced swelling, as the surface is more accessible towards hyaluronidases. Due to the sensitive application of hydrogels, in vivo studies regarding HA-SH stability and degradation of HA-SH hydrogels are from high interest. In general, chemical modification such as thiolation of HA should lead to a higher resistance against enzymatic degradation of the polymer [55]; however, biocompatibility of modified HA should not be impaired. Laffleur et al. [23] confirmed that chemical modification could have a great impact onto the stability as the thiolation process stabilized the polymer by intra- as well as inter-molecular disulfide bonds resulting in a higher resistance of the polymer towards degradation. Kafedjiiski et al. [14] investigated the viscous properties of different HA polymers (unmodified, thiolated and crosslinked thiolated) in presence of hyaluronidase as demonstrated in Figure 4. Within this study, it could be shown that HA is fastest degraded after addition of hyaluronidase, followed by thiolated HA with l-cysteine ethyl ester (HA-Cys), and finally, the crosslinked thiolated HA demonstrated only a minor decrease in viscosity due to the minimized excess of hyaluronidase onto HA. Moreover, Hahn et al. [33] found out that thiolated HA hydrogels are degraded only after 2 weeks in vivo. Additionally, HA hydrogels without disulfide bonds possessing other crosslinkage were tested resulting in a higher stability, which might be due to the absence of a thiol-exchange reaction between reduced glutathione presented in nearly all human cells and the disulfide bonds of polymers. Hence, it could be concluded that in vitro and in vivo data are not always correlating precisely, as many factors could have an additional influence in vivo. Degradation properties, viscosity and gelation time could be adjusted by varying the pH of the reaction mixture, the concentration of HA-SH and the stoichiometric ratio of thiol to acryloyl groups during the Michael addition [28]. Therefore, Dubbini et al. [52] examined gelation kinetic, mechanical properties and the swelling/degradation profile of vinyl sulfone thiolated HA hydrogels. Within this study, the degree of vinyl sulfonation and the degree of thiolation were shown to have an impact on the aforementioned properties. Another study from Li et al. [15] showed that HA-cysteine exhibited a protective effect for insulin against degradation by trypsin and α-chymotrypsin. This observation could be explained by the covalent attachment of the enzymes onto the polymer backbone by disulfide bond formation between the thiol moieties of HA-SH and the cysteine residues of the aforementioned proteases. This inhibitory effect could not be determined for unmodified hyaluronic acid. Furthermore, Liu et al. [31] synthesized thiolated HA in order to formulate a hydrogel with the ability of free radical scavenging and degradation resistance. Hence, (−)-epigallocatechin-3-*O*-gallate was attached to thiolated HA resulting in an inhibition of free radicals as well as the ability of inhibiting hyaluronidases.

### 3.4. Biocompatibility

The biocompatibility represents an important issue within formulation development from the academic as well as the industrial point of view, as novel formulations should not cause adverse effects after administration. Within several studies from Laffleur and co-workers, thiolated HA did not affect cell viability of Caco-2 cells [16,19,20,21,22,23]. Moreover, other research groups demonstrated high cell viability on 3T3, RAW 264.7 and HMEC cells [67]. Thiolated HA did not show any cytotoxicity towards primary human fibroblasts either [59]. Moreover, another study showed an enhanced fibronectin absorption via increasing the degree of sulfonation [53]. This process allows more cells to adhere on the glycosaminoglycan surface. Additionally, the degree of thiolation was investigated illustrating that more thiolation is leading to a lower biocompatibility of glycosaminoglycans. Besides, metabolic activity and cell growth were found to be promoted up to a higher limit of thiolation degree. The importance of the chemical composition of hydrogels could be proven and additionally it is opening opportunities for a large field of applications. Nonetheless, it seems not to pose a problem finding the optimal composition for a biocompatible hydrogel, as Sabbieti et al. [54] verified the biocompatibility of vinyl sulfone thiolated HA hydrogels developed by Dubbini et al. [52] in vivo as well. Apart from that, biocompatibility could at the same time be improved via anti-fibronectin aptamer functionalization as studied by Galli et al. [68]. As lower molecular weight HA ensured maximum cell survival, the composition of hydrogels plays a crucial role in finding the right compounds for specific application.

### 3.5. Permeation Enhancing Properties

The permeation enhancement is mainly the result of reduced glutathione (GSH) mediated protein tyrosine phosphatase inhibition. In detail, the presence of GSH facilities the permeation of compounds across the mucosa. The thiol groups of the polymer convert the oxidized form of glutathione to reduced form after a thiol-exchange reaction [69]. In most cases, the permeation enhancement of HA-SH is the consequence of this aforementioned effect. The permeation rate of curcumin for instance was found to be 4.4-fold enhanced by utilizing thiolated HA compared to unmodified HA [24]. On the contrary, an insulin permeation study utilizing cell monolayer showed that the permeation rate of insulin from HA-SH gel was lower compared to the unmodified gel. In addition, an increasing amount of thiol groups on the polymer backbone led to slower permeation. Within this set-up, the formation of disulfide bonds between insulin and HA-SH outweighed the permeation enhancing effect of the thiolated polymer [15]. To prevent the oxidation of two free thiol groups, S-protection via 6-mercaptonicotinamide might be the key.

### 3.6. Controlled Release

**Figure 5:**
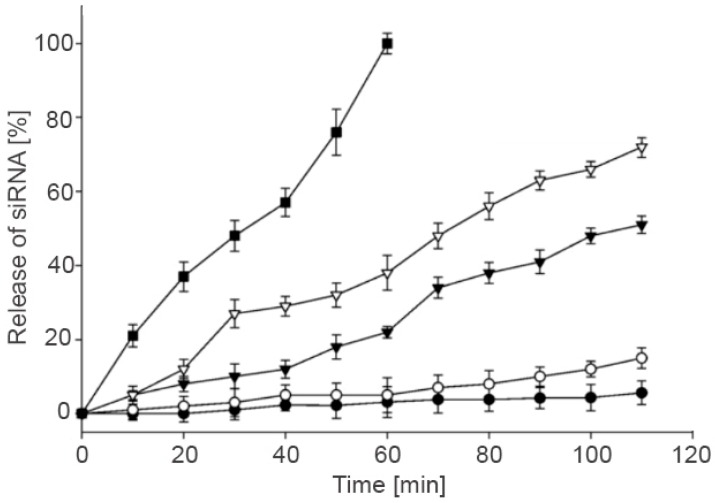
Release of siRNA from thiolated HA gels at various glutathione concentrations; (●) 0 mM GSH, (○) 0.1 mM GSH, (▼) 1 mM GSH, (▽) 5 mM GSH and (■) 10 mM GSH. Adapted from Lee et al. [29].

The release of active pharmaceutical ingredients incorporated in thiolated HA systems could be controlled by different parameters such as structural changes and pH. Censi et al. [49] developed in situ forming hydrogels via Tandem thermal gelling and Michael addition reaction utilized as carrier system for controlled peptide (bradykinin) release. This thiolated HA system represented high potential for peptide as well as protein drug delivery, as the release kinetic of bovine serum albumin containing gel could be tailored by altering the chemical structure of the polymer. Furthermore, another study suggested biocompatible, biodegradable and thiolated HA hydrogels for gene delivery [29]. Small interfering RNA (siRNA) was entrapped into thiolated HA hydrogel and successfully transported into the cells via CD44 receptors located on the surface of the cell. To gain better knowledge about the influence of GSH onto the siRNA release out of the hydrogel, different GSH concentrations were examined. Figure 5 displays that the release rate of siRNA out of the hydrogel depends on the GSH concentration in the buffer solution. At a GSH concentration of 10 mM the whole siRNA was released after 60 min, which indicated that the thiolated HA hydrogels are degraded more rapidly through increasing GSH concentrations. It could be assumed that these hydrogels are stable in extracellular conditions, as the extracellular compartment exhibits lower GSH concentration as applied during the release studies. As soon as the hydrogels reach the intracellular compartment the drug is likely released. Thus, thiolated HA hydrogels can be assessed as potent gene delivery systems exhibiting GSH dependent controlled release rate, such as against genetic disorders or cancer [30]. Han et al. for instance [70] developed core-crosslinked thiolated HA micelle for doxorubicin as potential targeted cancer therapy. Within this study, the core-crosslinkage led to sustained doxorubicin release, however, in presence of 10 mM GSH the release increased. As tumor cells have a high level of GSH and drug is released at the desired area, a controlled release as well as an improved therapeutic efficiency directly at the tumor site could be guaranteed. Fu et al. [56] demonstrated a novel dual pH- and reduction-responsive release system utilizing doxorubicin hydrochloride. An accelerated doxorubicin release could be determined at acidic pH (pH 5) and in presence of increasing GSH concentrations. A novel hydrogel depot for protein release was formulated by Yu and Chau [61], which was based on the De Gennes’ blob theory. This model represents concentrations by a sphere and the diameter is the average distance x between two chains. Furthermore, the diameter decreases as soon as the concertation increases. Altogether, the Gennes’ blob model describes polymer solutions, crosslinked polymers (gels), polymer welds, interfaces and polymers at surfaces. Within this study, two problems for vinyl sulfonate-thiol in situ hydrogel systems such as fast release and protein binding could be solved via increasing degree of modification and concentration of SH polymers. In addition, this crosslinked thiolated HA hydrogel was injected into rabbit eyes in order to achieve controlled release of bevacizumab [62]. This study resulted in a 107 times higher bevacizumab concentration compared to the bolus injection 6 months after injection. Another study demonstrated the controlled release of ovalbumin from the matrix of thiolated HA [18]. Thereby, thiolated HA showed improved stability against hyaluronidase, further this controlled release system could be identified as tunable release system through varying molecular weight of HA. Apart from this, Fan et al. [17] incorporated cationic liposomes onto the surface of thiolated HA in order to form nanoparticles with thiolated polyethylene glycols (PEG). These nanoparticles exhibited improved colloidal stability as well as a prolonged release of antigens. Recently, a review article from Campani et al. [71] described the potential of these lipid-based core shell nanoparticles, especially hyaluronic acid-based core-shell nanoparticles, concluding that these drug delivery systems are opening new therapeutic possibilities.

### 3.7. Further Applications of Thiolated HA

To accelerate the rejuvenation of tissues after injuries or damages, wound healing or regenerative medicine provides a tool for clinical practice. The combination of mucoadhesion and wound healing represents an interesting strategy. In particular, for healing of wounds on mucosal tissues the mucoadhesive properties of thiolated HA seem advantageous. Therefore, the adhesion of in vitro cultured and differentiated scaffold directly on the injured tissue with the ability of remaining at this area seems to highly on demand.

The utilization of thiolated HA offers numerous possibilities for engineering novel biomaterials. HA is present in the human body as the component of the extracellular matrix of the cartilage tissue or in the vitreous of the eye and it is part of the clinical practice for more than 35 years [72]. HA is the only non-sulfonated glycosaminoglycan and it is necessary for the stabilization of extracellular matrix, regulation of cell adhesion and mediation of cell proliferation. HA is continuously degraded within the body by hyaluronidase enzymes; thus, modifications of the polymer backbone such as thiolation are mandatory to obtain more mechanically and chemically robust materials [42]. For instance, Shu et al. [39] thiolated HA with dihydrazides generating HA-DTPH (dithiobis (propanoic dihydrazide)) and HA-DTBH (dithiobis (butyric dihydrazide)). The thiol groups were spontaneously oxidized on air and showed the ability to form hydrogel films via inter- and intramolecular disulfide bond formation. The use of these hydrogels with in vitro cultured fibroblasts led to proliferation even over 3 days. Moreover, HA-DTPH hydrogels were coupled to polyethylene glycols (PEG) to achieve an improved in situ crosslinkable HA hydrogel. The hydrogels with thiolated HA and PEGDA (PEG-diacrylate) demonstrated an in situ crosslinking and resulted in a 10-fold higher cell density over 4 weeks culture period compared to the initial cell density. Due to the in situ crosslinking of this polymer, hydrogel possessed high potential for wound healing and tissue repair. The same thiolated HA with PEGDA was modified utilizing peptides containing the Arg-Gly-Asp sequence to further endorse cell attachment, spreading and proliferation for novel injectable biomaterials. Due to the significant proliferation enhancement of Arg-Gly-Asp peptides, HA-DTPH-PEGDA hydrogels were identified as formulations with high potential for regenerative medicine in vivo [41]. Ouasti et al. [25] categorized HA/PEG hydrogels in the following groups: type I gels—HA dispersed in a PEGDA network, type II gels—HA-SH as chain transfer agent during PEGDA polymerization and type III gels—in situ preparation and polymerization of HA-SA/PEGDA macromolecules. Within this study, a relation for type II gel between fibroblast spreading and the mechanical properties was found out, which may open novel possibilities of cell and material fine tuning. As unmodified HA hydrogels are attached on the surface of cells and proliferate in a 2D model, the fabrication of 3D scaffolds based on electrospinning is of great interest. For instance, Ji et al. [43,44] demonstrated fibroblasts migrating and forming 3D dendritic morphology inside the HA-DTPH nanofibrous scaffold. These nanofibers possessed the potential for cell encapsulation and 3D cell culturing for tissue regeneration. Furthermore, these nanofibers exhibited the ability of controlling cell adhesion as well as cell morphology [73]. As previously mentioned, regenerative medicine for the treatment of neuronal injuries could revolutionize the medical therapy. In this field, Horn et al. [45] illustrated that HA-DTPH-PEGDA hydrogels might have a high potential for neurite outgrowth. A 50% increase in neurite length compared to the fibrin samples was achieved. In addition, regenerative medicine is of great importance in heart regeneration; namely, replacing cardiovascular implants with bioprinting of vessel-like constructs utilizing HA hydrogels can further improve the quality of life [50]. Besides, Young and Engel [58] demonstrated that hydrogels enhance the differentiation of cardiomyocytes in vitro. Thereby, pre-cardiac cells grown on collagen-coated HA showed 60% increased maturing muscle fibers as well as 3-fold higher number of cardiac-specific markers over a time period of 2 weeks.

**Figure 6:**
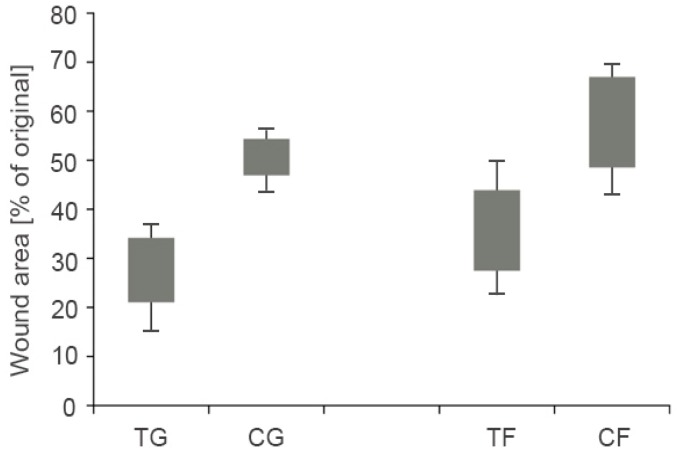
Overview of the wound area comparing thiolated carboxymethyl HA film and gel studied on rats. Wound area calculated in percent referring to the original wound area. Gel (TG) and film (TF) treatments compared with untreated controls (CG and CF) on day 7 after wounding. Bars represent 95% confidence intervals of the means; lines represent the range of values obtained. Adapted from Yang et al. [35].

The treatment or accelerated healing of corneal injuries as demonstrated by Yang et al. [48] illustrated the wound healing properties of a thiol-modified and crosslinked HA hydrogel by abrasion and alkali burning of the coronal epithelium from rabbits. Within this study, 1% of the thiolated HA hydrogel formulation was applied on the right eye four times per day resulting in a closure of coronal wound in the abrasion model. Furthermore, the closure rate as well as the thickness regarding the alkali burn model was greater compared to the control. Since more than 161 million people worldwide suffer from visual impairment and thereof 37 million are blind, an adequate treatment is of high interest [74]. Generally, coronial blindness can be surgical treated by transplantation of the corona. As 90% of visually impaired people live in low- or middle-income countries, a treatment such as Espandar et al. [75] demonstrated would be highly beneficial. Within this study, HyStem^®^—a hydrogel kit containing thiol-modified hyaluronan, thiol-reactive PEGDA crosslinker and thiol-modified collagen resulting in a semisynthetic extracellular matrix, which represents an affordable clinical product [76]—showed the highest yield of human adipose-driven stem cells (h-ADSCs) compared to other HA-derived synthetic extracellular matrix culture media [77]. Usually, h-ADSC, representing a derived synthetic extracellular matrix, are utilized to identify the capacity of proliferation and survival of cells [75]. More importantly, HyStem^®^ hydrogels tested via h-ADSC can serve as carrier for stem cell-based therapy and for tissue engineering [78]. Moreover, HyStem^®^ has an effect on controlling the retinal progenitor cell being part of the retinal repair, as it is forming a microenvironment for self-renewal and differentiation of retinal progenitor cell [79]. Zarembinski et al. [80] confirmed that hydrogels such as HyStem^®^ are supporting the 3D culturing of h-ADSCs in vitro and especially in vivo demonstrating high biocompatibility. h-ADSCs were in situ encapsulated in PEG hyperbranched Hystem^®^ hydrogels resulting in a high cell viability over 7 days, which might be a helpful tool for wound healing in the near future [81]. Besides, Yang et al. [35] utilized thiolated carboxymethyl HA films to test the efficiency of wound healing across multiple species, such as rats, dogs and horses. An increased keratinocyte proliferation resulting in thicker epidermis compared to the control could be observed. A significant (*p* = 0.001) difference between the wound area of the gel and the untreated control could be observed, as displayed in Figure 6. The wound area of the film treated group was smaller compared to the control (*p* = 0.01). Moreover, even vocal fold scars causing dysphonia could demonstrate significantly improved biomedical properties regarding elasticity and viscosity via a hydrogel kit containing equal ingredients such as Hystem^®^ [57]. Walimbe et al. [82] recently summarized the advantages of thiolated HA hydrogels in order to engineer vocal fold tissue.

## 4. Delivery Systems

### 4.1. Buccal Drug Delivery

Buccal drug delivery represents an appropriate alternative to the most common route namely the oral administration. Via the buccal route degradation in the stomach and small intestine can be avoided, as well the hepatic first-pass metabolism can be bypassed. The buccal mucosa is suitable for controlled drug delivery for extended time periods [83,84,85,86]. Nevertheless, lot of diseases could be treated in an improved way via the buccal route; just mentioning an example, patients suffering from Parkinson disease are confronted with dysphagia and therefore, the application of transmucosal buccal delivery systems might overcome this hindrance [23]. As for this administration high mucoadhesion is mandatory, thiolated or preactivated thiolated HA offers an effective tool to guarantee a prolonged residence time of the drug on the mucosa. For instance, our research group demonstrated a 4-fold improved mucoadhesion time of thiolated HA preactivated with 6-mercaptonicotinamide compared to unmodified HA that makes HA-SH as well as preactivated HA-SH a suitable polymer for buccal delivery [19]. Preactivated HA showed high stability as well as no cytotoxicity on Caco-2 cells. The preactivation or S-protection is of great importance in order to guarantee efficient stability against oxidation prior to administration and via preactivation of HA-SH an even longer residence time is predictable [16].

### 4.2. Vaginal Drug Delivery

Vaginal drug delivery is offering numerous of advantages, namely easy access, painlessness and prolonged retention of formulation, as no movements such as in the gastrointestinal tract are required [87]. The vagina possesses a highly beneficial permeation area, high vascularization, low enzymatic activity and most importantly, drugs applied on the vaginal site bypass the first-pass effect [88]. Through the application of mucoadhesive thiolated HA the retention time of drug delivery systems could be tremendously improved and subsequently the drug contact time at the target site is prolonged [89,90]. Nowak et al. [20] thiolated HA with l-cysteine ethyl ester followed by a preactivation with 6-mercaptonicotinamide. Preactivated HA demonstrated a 3.6-fold prolonged disintegration time compared to unmodified HA as well as a prolonged mucoadhesion time, presumably leading to an increased contact time of the drug with the adsorption sites in vivo. Moreover, these modified HAs were found to be biocompatible and therefore they are safe tools for vaginal application. Agrahari et al. [60] performed an interesting study based on nanofibers of thiolated HA loaded with tenofovir, which were further characterized in vitro and in vivo for topical intravaginal delivery of HIV/AIDS microbicides. Thereby, the nanofibers were found to be non-toxic and no damage on the genital tract of mice could be observed. Within the vaginal tissue, a significant bioavailability as well as retention improvement of tenofovir could be identified compared to the 1% tenofovir gel.

### 4.3. Ocular Drug Delivery

Due to its high biocompatibility, HA-SH might be applied onto the ocular surface as well. For example, Williams and Mann [36] demonstrated high effectiveness of crosslinked and thiolated HA hydrogels on dry eye syndrome in vivo. After application of the polymer in rabbits an increase in tear breakup time was found as well as the stabilization of the tear film was observed. In vivo studies on dogs suffering from keratoconjunctivitis sicca showed significantly reduces symptoms. Based on these results, Williams and Mann [37] performed a masked, randomized clinical study in dogs with keratoconjunctivitis sicca. Within this study, crosslinked and thiolated HA hydrogels showed significantly improved ocular surface health compared to HA-based iDrop^®^ Vet Plus Eye Lubricant (ITRD; I-MED Animal Health). In another study, thiolated HA hydrogels were investigated regarding improved retention via 3D computational finite eye models. Simple geometrical modifications had an impact onto the hydrogel film retention [38]. The application of contact lenses generally contributes to the exacerbation of the symptoms associated to the dry eye syndrome. Reflexing to this, Korogiannaki et al. [32] formulated a thiolated HA hydrogel for the improvement of surface properties of contact lens, which improve a higher lens compatibility with the ocular environment. Apart from dry eye syndrome, improving the treatment of retinal attachment represents high industrial interest, as the current vitreous substitute such as silicone oils are not biodegradable and have to be removed via a second surgery. The efficiency of two artificial vitreous body substitutes (VBS) was evaluated and compared to silicone oil as gold standard. Therefore, the retina of rabbits was at first reattached via the injection of air and thereafter, the eye was filled with silicon oil or the corresponding number of hydrogels. Within the silicon oil group 87.5% demonstrated a recovered retinal attachment, whereas 73.3% of the hydrogel group exhibited intact retina. Therefore, biodegradable thiolated HA hydrogels show a comparable efficiency compared to the gold standard with the possibility of avoiding a second operation [91].

## 5. Product Development

As thiolated HA has great potential within the medical field, some products are already available on the market. For instance, Glycosil^®^ [92] represents a thiol-modified hyaluronic acid and is utilized for 3D cell culturing and tissue engineering applications [93]. HyStem^®^, a hydrogel kit containing Glycosil^®^ as a main component, could be applied for biomedical applications such as wound healing as mentioned previously [76]. Moreover, a fast-gelling thiolated HA was developed from Vornia Biomaterials in collaboration with the University College Dublin, Ireland. This thiolated HA possesses a purity of 98%, a gelation time of less than 30 min [94]. Apart from hydrogel kits, Croma-Pharma GmbH completed a Phase I study in April 2016 aiming the safety of implanting thiolated HA in patients suffering from primary open angle glaucoma [95]. In general, thiolated HA occupies an improved mucoadhesiveness and cohesive capacity on the ocular surface compared to not thiolated HA. The residence time is improved, and the administration interval is reduced. In addition, Eyegate Pharmaceuticals, Inc. has an ocular bandage gel consisting of thiolated carboxymethyl HA for ocular wound healing in its pipeline [96]. Therefore, it could be assumed that mucoadhesive thiolated HA will attract lots of attention in the imminent years.

## 6. Conclusions

The thiolation of hyaluronic acid leads to a higher stability and enhanced mucoadhesive properties of the polymeric backbone. Due to its versatility, thiolated hyaluronic acids can be utilized for wide ranges of pharmaceutical application. As exhibiting beneficial toxicological properties, it can be used beside oral application on sensitive target sites as well, such as vaginal or ocular surface. Furthermore, due to its in situ gelling properties it is a potent tool for mucoadhesive hydrogel films possessing controlled release properties. The properties of the forming gels may be easily tailored by adjusting the chemical properties, the density of the ligands and crosslinking rate. In addition, HA-SH is a promising excipient for wound healing, as it promotes the proliferation of cells by providing an appropriate 3D microenvironment. It is highly predictable, that within the next years HA-SH will gain more attention due to the rising relevance for targeted mucoadhesive drug delivery, cell-based therapy systems and wound healing. As thiolated HA is a biodegradable polymer, it has great potential for biomedical applications, for instance on eyes, to improve patient compliance and to save medical expenses. Finally, thiolated HA is in the pipeline of pharmaceutical industry, and therefore some highly innovative products will enter the market soon.

## Figures and Tables

**Table 1:** Overview various thiolated HAs including type of thiolated HA, targeted chemical group of HA, ligand and reaction type.

Type of thiolated HA	Targeted chemical group of HA	Ligand	Reaction type	Reference
HA-SH	Carboxylic	l-Cysteine	Amidation	[15,16,17,18]
HA-SH	Carboxylic	l-Cysteine ethyl ester	Amidation	[14,19,20,21,22,23,24]
HA-SH	Carboxylic	Cysteamine	Amidation	[25,26,27,28]
HA-SH	Carboxylic	Cysteamine	Amidation	[29,30,31,32]
HA(-ADH)-SH	Carboxylic	1. Adipic acid dihydrazide (ADH) 2. 2-Immoinothiolane	1. Amidation 2. Thiolation with Traut’s reagent	[33,34]
Thiolated carboxymethyl HA	Carboxylic	5,5′-Dithiobis (2-nitrobenzoic acid)	Amidation	[35,36,37,38]
HA-DTPH	Carboxylic	Dithiobis (propanoic dihydrazide)	Amidation	[39,40,41,42,43,44,45,46,47,48,49,50,51,52,53,54,55,56,57]
HA-DTBH	Carboxylic	Dithiobis (butyric dihydrazide)	Amidation	[39]
HA-PDPH	Carboxylic	3-(2-pyridyldithio) propionyl hydrazide	Amidation	[58]
HA-SH	Hydroxyl	Ethylene sulfide	Ether formation	[59,60]
HA-SH	Hydroxyl	1. Divinyl sulfone 2. Dithiothreitol	1. Ether formation 2. Amidation	[61,62]

## References

[1] Schanté C.E., Zuber G., Herlin C., Vandamme T.F. (2011). Chemical modifications of hyaluronic acid for the synthesis of derivatives for a broad range of biomedical applications. Carbohydr. Polym..

[2] Bernkop-Schnürch A. (2000). Muco-Adhesive Polymers, Use Thereof and Method For Producting the Same. Patent Family.

[3] Prestwich G.D. (2011). Hyaluronic acid-based clinical biomaterials derived for cell and molecule delivery in regenerative medicine. J. Control. Release.

[4] Iqbal J., Shahnaz G., Dünnhaupt S., Müller C., Hintzen F., Bernkop-Schnürch A. (2012). Preactivated thiomers as mucoadhesive polymers for drug delivery. Biomaterials.

[5] Wirostko B., Mann B.K., Williams D.L., Prestwich G.D. (2014). Ophthalmic Uses of a Thiol-Modified Hyaluronan-Based Hydrogel. Adv. Wound Care (New Rochelle).

[6] Nyström B., Kjøniksen A.L., Beheshti N., Maleki A., Zhu K., Knudsen K.D., Pamies R., Hernández Cifre J.G., García de la Torre J. (2010). Characterization of polyelectrolyte features in polysaccharide systems and mucin. Adv. Colloid Interface Sci..

[7] Day A.J., Sheehan J.K. (2001). Hyaluronan: Polysaccharide chaos to protein organisation. Curr. Opin. Struct. Biol..

[8] Fraser J.R., Laurent T.C., Laurent U.B. (1997). Hyaluronan: Its nature, distribution, functions and turnover. J. Intern. Med..

[9] Robert L., Robert A.M., Renard G. (2010). Biological effects of hyaluronan in connective tissues, eye, skin, venous wall. Role in aging. Pathol. Biol. (Paris).

[10] Brown T.J., Laurent U.B., Fraser J.R. (1991). Turnover of hyaluronan in synovial joints: Elimination of labelled hyaluronan from the knee joint of the rabbit. Exp. Physiol..

[11] Aruffo A., Stamenkovic I., Melnick M., Underhill C.B., Seed B. (1990). CD44 is the principal cell surface receptor for hyaluronate. Cell.

[12] Stern R., Kogan G., Jedrzejas M.J., Soltés L. (2007). The many ways to cleave hyaluronan. Biotechnol. Adv..

[13] Price R.D., Berry M.G., Navsaria H.A. (2007). Hyaluronic acid: The scientific and clinical evidence. J. Plast. Reconstr. Aesthet. Surg..

[14] Kafedjiiski K., Jetti R.K., Föger F., Hoyer H., Werle M., Hoffer M., Bernkop-Schnürch A. (2007). Synthesis and in vitro evaluation of thiolated hyaluronic acid for mucoadhesive drug delivery. Int. J. Pharm..

[15] Li X., Yu G., Jin K., Yin Z. (2012). Hyaluronic acid l-cysteine conjugate exhibits controlled-release potential for mucoadhesive drug delivery. Pharmazie.

[16] Pereira de Sousa I., Suchaoin W., Zupančič O., Leichner C., Bernkop-Schnürch A. (2016). Totally S-protected hyaluronic acid: Evaluation of stability and mucoadhesive properties as liquid dosage form. Carbohydr. Polym..

[17] Fan Y., Sahdev P., Ochyl L.J., Akerberg J.J., Moon J.J. (2015). Cationic liposome-hyaluronic acid hybrid nanoparticles for intranasal vaccination with subunit antigens. J. Control. Release.

[18] Du J., Fu F., Shi X., Yin Z. (2015). Controlled release of a model protein drug ovalbumin from thiolated hyaluronic acid matrix. J. Drug Deliv. Sci. Technol..

[19] Laffleur F., Röggla J., Idrees M.A., Griessinger J. (2014). Chemical modification of hyaluronic acid for intraoral application. J. Pharm. Sci..

[20] Nowak J., Laffleur F., Bernkop-Schnürch A. (2015). Preactivated hyaluronic acid: A potential mucoadhesive polymer for vaginal delivery. Int. J. Pharm..

[21] Laffleur F., Wagner J., Mahmood A. (2015). In vitro and ex vivo evaluation of biomaterials’ distinctive properties as a result of thiolation. Future Med. Chem..

[22] Laffleur F., Psenner J., Suchaoin W. (2015). Permeation enhancement via thiolation: In vitro and ex vivo evaluation of hyaluronic acid-cysteine ethyl ester. J. Pharm. Sci..

[23] Laffleur F., Wagner J., Barthelmes J. (2015). A potential tailor-made hyaluronic acid buccal delivery system comprising rotigotine for Parkinson’s disease?. Future Med. Chem..

[24] Laffleur F., Schmelzle F., Ganner A., Vanicek S. (2017). In Vitro and Ex Vivo Evaluation of Novel Curcumin-Loaded Excipient for Buccal Delivery. AAPS PharmSciTech.

[25] Ouasti S., Donno R., Cellesi F., Sherratt M.J., Terenghi G., Tirelli N. (2011). Network connectivity, mechanical properties and cell adhesion for hyaluronic acid/PEG hydrogels. Biomaterials.

[26] Bian S., He M., Sui J., Cai H., Sun Y., Liang J., Fan Y., Zhang X. (2016). The self-crosslinking smart hyaluronic acid hydrogels as injectable three-dimensional scaffolds for cells culture. Colloids Surf. B Biointerfaces.

[27] Ding J., He R., Zhou G., Tang C., Yin C. (2012). Multilayered mucoadhesive hydrogel films based on thiolated hyaluronic acid and polyvinylalcohol for insulin delivery. Acta Biomater..

[28] Liu Y., Zhang F., Ru Y. (2015). Hyperbranched phosphoramidate-hyaluronan hybrid: A reduction-sensitive injectable hydrogel for controlled protein release. Carbohydr. Polym..

[29] Lee H., Mok H., Lee S., Oh Y.K., Park T.G. (2007). Target-specific intracellular delivery of siRNA using degradable hyaluronic acid nanogels. J. Control. Release.

[30] Yin T., Liu J., Zhao Z., Dong L., Cai H., Yin L., Zhou J., Huo M. (2016). Smart nanoparticles with a detachable outer shell for maximized synergistic antitumor efficacy of therapeutics with varying physicochemical properties. J. Control. Release.

[31] Liu C., Bae K.H., Yamashita A., Chung J.E., Kurisawa M. (2017). Thiol-Mediated Synthesis of Hyaluronic Acid-Epigallocatechin-3-*O*-Gallate Conjugates for the Formation of Injectable Hydrogels with Free Radical Scavenging Property and Degradation Resistance. Biomacromolecules.

[32] Korogiannaki M., Zhang J., Sheardown H. (2017). Surface modification of model hydrogel contact lenses with hyaluronic acid via thiol-ene “click” chemistry for enhancing surface characteristics. J. Biomater. Appl..

[33] Hahn S.K., Park J.K., Tomimatsu T., Shimoboji T. (2007). Synthesis and degradation test of hyaluronic acid hydrogels. Int. J. Biol. Macromol..

[34] Hahn S.K., Kim J.S., Shimobouji T. (2007). Injectable hyaluronic acid microhydrogels for controlled release formulation of erythropoietin. J. Biomed. Mater. Res. A.

[35] Yang G., Prestwich G.D., Mann B.K. (2011). Thiolated carboxymethyl-hyaluronic-acid-based biomaterials enhance wound healing in rats, dogs, and horses. ISRN Vet. Sci..

[36] Williams D.L., Mann B.K. (2013). A Crosslinked HA-Based Hydrogel Ameliorates Dry Eye Symptoms in Dogs. Int. J. Biomater..

[37] Williams D.L., Mann B.K. (2014). Efficacy of a crosslinked hyaluronic acid-based hydrogel as a tear film supplement: A masked controlled study. PLoS ONE.

[38] Colter J., Wirostko B., Coats B. (2018). Finite Element Design Optimization of a Hyaluronic Acid-Based Hydrogel Drug Delivery Device for Improved Retention. Ann. Biomed. Eng..

[39] Shu X.Z., Liu Y., Luo Y., Roberts M.C., Prestwich G.D. (2002). Disulfide cross-linked hyaluronan hydrogels. Biomacromolecules.

[40] Liu Y., Shu X.Z., Gray S.D., Prestwich G.D. (2004). Disulfide-crosslinked hyaluronan-gelatin sponge: Growth of fibrous tissue in vivo. J. Biomed. Mater. Res. A.

[41] Shu X.Z., Ghosh K., Liu Y., Palumbo F.S., Luo Y., Clark R.A., Prestwich G.D. (2004). Attachment and spreading of fibroblasts on an RGD peptide-modified injectable hyaluronan hydrogel. J. Biomed. Mater. Res. A.

[42] Shu X.Z., Ahmad S., Liu Y., Prestwich G.D. (2006). Synthesis and evaluation of injectable, in situ crosslinkable synthetic extracellular matrices for tissue engineering. J. Biomed. Mater. Res. A.

[43] Ji Y., Ghosh K., Shu X.Z., Li B., Sokolov J.C., Prestwich G.D., Clark R.A., Rafailovich M.H. (2006). Electrospun three-dimensional hyaluronic acid nanofibrous scaffolds. Biomaterials.

[44] Ji Y., Ghosh K., Li B., Sokolov J.C., Clark R.A., Rafailovich M.H. (2006). Dual-syringe reactive electrospinning of cross-linked hyaluronic acid hydrogel nanofibers for tissue engineering applications. Macromol. Biosci..

[45] Horn E.M., Beaumont M., Shu X.Z., Harvey A., Prestwich G.D., Horn K.M., Gibson A.R., Preul M.C., Panitch A. (2007). Influence of cross-linked hyaluronic acid hydrogels on neurite outgrowth and recovery from spinal cord injury. J. Neurosurg. Spine.

[46] Orlandi R.R., Shu X.Z., McGill L., Petersen E., Prestwich G.D. (2007). Structural variations in a single hyaluronan derivative significantly alter wound-healing effects in the rabbit maxillary sinus. Laryngoscope.

[47] Vanderhooft J.L., Alcoutlabi M., Magda J.J., Prestwich G.D. (2009). Rheological Properties of Cross-Linked Hyaluronan–Gelatin Hydrogels for Tissue Engineering. Macromol. Biosci..

[48] Yang G., Espandar L., Mamalis N., Prestwich G.D. (2010). A cross-linked hyaluronan gel accelerates healing of corneal epithelial abrasion and alkali burn injuries in rabbits. Vet. Ophthalmol..

[49] Censi R., Fieten P.J., di Martino P., Hennink W.E., Vermonden T. (2010). In situ forming hydrogels by tandem thermal gelling and Michael addition reaction between thermosensitive triblock copolymers and thiolated hyaluronan. Macromolecules.

[50] Skardal A., Zhang J., Prestwich G.D. (2010). Bioprinting vessel-like constructs using hyaluronan hydrogels crosslinked with tetrahedral polyethylene glycol tetracrylates. Biomaterials.

[51] Horkay F., Magda J., Alcoutlabi M., Atzet S., Zarembinski T. (2010). Structural, mechanical and osmotic properties of injectable hyaluronan-based composite hydrogels. Polymer.

[52] Dubbini A., Censi R., Butini M.E., Sabbieti M.G., Agas D., Vermonden T., Di Martino P. (2015). Injectable hyaluronic acid/PEG-p(HPMAm-lac)-based hydrogels dually cross-linked by thermal gelling and Michael addition. Eur. Polym. J..

[53] Köwitsch A., Niepel M.S., Michanetzis G.P., Missirlis Y.F., Groth T. (2016). Effect of Immobilized Thiolated Glycosaminoglycans on Fibronectin Adsorption and Behavior of Fibroblasts. Macromol. Biosci..

[54] Sabbieti M.G., Dubbini A., Laus F., Paggi E., Marchegiani A., Capitani M., Marchetti L., Dini F., Vermonden T., Di Martino P. (2016). In vivo biocompatibility of p(HPMAm-lac)-PEG hydrogels hybridized with hyaluronan. J. Tissue Eng. Regen. Med..

[55] Liu Y., Zheng X., Shu G.D. (2005). Prestwich, Biocompatibility and stability of disulfide-crosslinked hyaluronan films. Biomaterials.

[56] Fu C., Li H., Li N., Miao X., Xie M., Du W., Zhang L.M. (2015). Conjugating an anticancer drug onto thiolated hyaluronic acid by acid liable hydrazone linkage for its gelation and dual stimuli-response release. Carbohydr. Polym..

[57] Thibeault S.L., Klemuk S.A., Chen X., Quinchia Johnson B.H. (2011). In Vivo engineering of the vocal fold ECM with injectable HA hydrogels-late effects on tissue repair and biomechanics in a rabbit model. J. Voice.

[58] Young J.L., Engler A.J. (2011). Hydrogels with time-dependent material properties enhance cardiomyocyte differentiation in vitro. Biomaterials.

[59] Serban M.A., Yang G., Prestwich G.D. (2008). Synthesis, characterization and chondroprotective properties of a hyaluronan thioethyl ether derivative. Biomaterials.

[60] Agrahari V., Meng J., Ezoulin M.J., Youm I., Dim D.C., Molteni A., Hung W.T., Christenson L.K., Youan B.C. (2016). Stimuli-sensitive thiolated hyaluronic acid based nanofibers: Synthesis, preclinical safety and in vitro anti-HIV activity. Nanomedicine.

[61] Yu Y., Chau Y. (2015). Formulation of in situ chemically cross-linked hydrogel depots for protein release: From the blob model perspective. Biomacromolecules.

[62] Yu Y., Lau L.C., Lo A.C., Chau Y. (2015). Injectable Chemically Crosslinked Hydrogel for the Controlled Release of Bevacizumab in Vitreous: A 6-Month In Vivo Study. Transl. Vis. Sci. Technol..

[63] Oh S., Wilcox M., Pearson J.P., Borrós S. (2015). Optimal design for studying mucoadhesive polymers interaction with gastric mucin using a quartz crystal microbalance with dissipation (QCM-D): Comparison of two different mucin origins. Eur. J. Pharm. Biopharm..

[64] Bernkop-Schnürch A., Hornof M., Zoidl T. (2003). Thiolated polymers—Thiomers: Synthesis and in vitro evaluation of chitosan-2-iminothiolane conjugates. Int. J. Pharm..

[65] Salamat-Miller N., Chittchang M., Johnston T.P. (2005). The use of mucoadhesive polymers in buccal drug delivery. Adv. Drug Deliv. Rev..

[66] Shinkar D.M., Dhake A.S., Setty C.M. (2012). Drug delivery from the oral cavity: A focus on mucoadhesive buccal drug delivery systems. PDA J. Pharm. Sci. Technol..

[67] Pedrosa S.S., Pereira P., Correia A., Moreira S., Rocha H., Gama F.M. (2016). Biocompatibility of a Self-Assembled Crosslinkable Hyaluronic Acid Nanogel. Macromol. Biosci..

[68] Galli C., Parisi L., Piergianni M., Smerieri A., Passeri G., Guizzardi S., Costa F., Lumetti S., Manfredi E., Macaluso G.M. (2016). Improved scaffold biocompatibility through anti-Fibronectin aptamer functionalization. Acta Biomater..

[69] Clausen A.E., Kast C.E., Bernkop-Schnürch A. (2002). The role of glutathione in the permeation enhancing effect of thiolated polymers. Pharm. Res..

[70] Han H.S., Choi K.Y., Ko H., Jeon J., Saravanakumar G., Suh Y.D., Lee D.S., Park J.H. (2015). Bioreducible core-crosslinked hyaluronic acid micelle for targeted cancer therapy. J. Control. Release.

[71] Campani V., Giarra S., de Rosa G. (2018). Lipid-based core-shell nanoparticles: Evolution and potentialities in drug delivery. OpenNano.

[72] Burdick J.A., Prestwich G.D. (2011). Hyaluronic Acid Hydrogels for Biomedical Applications. Adv. Mater..

[73] Wade R.J., Bassin E.J., Gramlich W.M., Burdick J.A. (2015). Nanofibrous hydrogels with spatially patterned biochemical signals to control cell behavior. Adv. Mater..

[74] World Health Organization. http://www.who.int/features/factfiles/vision/01_en.html.

[75] Espandar L., Bunnell B., Wang G.Y., Gregory P., McBride C., Moshirfar M. (2012). Adipose-derived stem cells on hyaluronic acid-derived scaffold: A new horizon in bioengineered cornea. Arch. Ophthalmol..

[76] ESIBIO Stem Cell Solutions. http://www.esibio.com/index.php/products/product-category/hydrogels-kits/hystem-hydrogels/.

[77] Prestwich G.D., Erickson I.E., Zarembinski T.I., West M., Tew W.P. (2012). The translational imperative: Making cell therapy simple and effective. Acta Biomater..

[78] Gwon K., Kim E., Tae G. (2017). Heparin-hyaluronic acid hydrogel in support of cellular activities of 3D encapsulated adipose derived stem cells. Acta Biomater..

[79] Liu Y., Wang R., Zarembinski T.I., Doty N., Jiang C., Regatieri C., Zhang X., Young M.J. (2013). The application of hyaluronic acid hydrogels to retinal progenitor cell transplantation. Tissue Eng. Part A.

[80] Zarembinski T.I., Doty N.J., Erickson I.E., Srinivas R., Wirostko B.M., Tew W.P. (2014). Thiolated hyaluronan-based hydrogels crosslinked using oxidized glutathione: An injectable matrix designed for ophthalmic applications. Acta Biomater..

[81] Hassan W., Dong Y., Wang W. (2013). Encapsulation and 3D culture of human adipose-derived stem cells in an in-situ crosslinked hybrid hydrogel composed of PEG-based hyperbranched copolymer and hyaluronic acid. Stem Cell Res. Ther..

[82] Walimbe T., Panitch A., Sivasankar P.M. (2017). A Review of Hyaluronic Acid and Hyaluronic Acid-based Hydrogels for Vocal Fold Tissue Engineering. J. Voice.

[83] Bernkop-Schnürch A., Dünnhaupt S. (2012). Chitosan-based drug delivery systems. Eur. J. Pharm. Biopharm..

[84] Hauptstein S., Hintzen F., Müller C., Ohm M., Bernkop-Schnürch A. (2014). Development and in vitro evaluation of a buccal drug delivery system based on preactivated thiolated pectin. Drug Dev. Ind. Pharm..

[85] Laffleur F., Fischer A., Schmutzler M., Hintzen F., Bernkop-Schnürch A. (2015). Evaluation of functional characteristics of preactivated thiolated chitosan as potential therapeutic agent for dry mouth syndrome. Acta Biomater..

[86] Laffleur F., Shahnaz G., Islambulchilar Z., Bernkop-Schnürch A. (2013). Design and in vitro evaluation of a novel polymeric excipient for buccal applications. Future Med. Chem..

[87] Hombach J., Palmberger T.F., Bernkop-Schnürch A. (2009). Development and in vitro evaluation of a mucoadhesive vaginal delivery system for nystatin. J. Pharm. Sci..

[88] Friedl H.E., Dünnhaupt S., Waldner C., Bernkop-Schnürch A. (2013). Preactivated thiomers for vaginal drug delivery vehicles. Biomaterials.

[89] Baloglu E., Ay Senyıgıt Z., Karavana S.Y., Vetter A., Metın D.Y., Hilmioglu Polat S., Guneri T., Bernkop-Schnurch A. (2011). In vitro evaluation of mucoadhesive vaginal tablets of antifungal drugs prepared with thiolated polymer and development of a new dissolution technique for vaginal formulations. Chem. Pharm. Bull. (Tokyo).

[90] Baloglu E., Senyigit Z.A., Karavana S.Y., Bernkop-Schnürch A. (2009). Strategies to prolong the intravaginal residence time of drug delivery systems. J. Pharm. Pharm. Sci..

[91] Schnichels S., Schneider N., Hohenadl C., Hurst J., Schatz A., Januschowski K., Spitzer M.S. (2017). Efficacy of two different thiol-modified crosslinked hyaluronate formulations as vitreous replacement compared to silicone oil in a model of retinal detachment. PLoS ONE.

[92] ESIBIO Stem Cell Solutions. http://www.esibio.com/index.php/products/popular-brands/glycosil/glycosil-hyaluronic-acid/.

[93] Bi X., Liang A., Tan Y., Maturavongsadit P., Higginbothem A., Gado T., Gramling A., Bahn H., Wang Q. (2016). Thiol-ene crosslinking polyamidoamine dendrimer-hyaluronic acid hydrogel system for biomedical applications. J. Biomater. Sci. Polym. Ed..

[94] VORNIA Biomaterials. http://www.vornia.com/products-page/functionalized-biopolymers/thiol-modified-hyaluronic-acid/.

[95] ClinicalTrials.gov. https://clinicaltrials.gov/ct2/show/NCT01887873?term=Croma+pharma&draw=2&rank=8.

[96] Eyegate Pharmaceuticals. http://www.eyegatepharma.com/pipeline/ocular-bandage-gel/.

